# The Obstetric Training of the Medical Student

**Published:** 1920-06-12

**Authors:** 


### THE OBSTETRIC TRAINING OF THE MEDICAL STUDENT.

At the meeting of the General Medical Council on June 3 a report was submitted by the Education Committee on the teaching of practical midwifery; and the Committee's recommendations were, with trifling modifications, adopted by the Council. The subject is one of the greatest importance, and it is highly satisfactory to realise that the General Medical Council is doing its best to remedy the haphazard and often unsatisfactory nature of the training which medical students at present receive in practical midwifery. Some improvement does seem to have occurred recently, but there is still room for a great deal more; and although the proposals adopted by the Council are not all entirely beyond controversion and are not likely to bear immediate fruit, yet they are in the main to be approved as steps along the path of progress.

To realise how unsatisfactory much of the training of the medical student—the medical practitioner of to-morrow—has been at most centres of medical education, it must be recalled that this training cannot by the nature of things be tested at qualifying examinations; and that to a great extent it has to be guaranteed merely by certificates of attendance on courses of lectures and on the conduct of a minimum number of cases of labour. Schools can insist on attendance at lectures, but they cannot enforce attention to the lecturer. The conduct of twenty cases of labour, supervised by a keen and competent teacher, can lay the foundation of a high degree of proficiency in obstetrics; but in practice the teachers are only too often themselves inexperienced and their supervision extremely perfunctory. Not through any fault, necessarily, of either student or supervisor, this clinical training is absolutely wasted in far too many cases. The Council doubtless recognises this when it affirms its belief that "the teaching of midwifery can never be satisfactory until students receive it at the bedside and until it is as thorough and practical as that of surgery; and that such teaching will only be possible when large lying-in hospitals are available throughout the country." The Council is therefore begging the Government departments concerned that existing Poor-law lying-in institutions be utilised for clinical instruction, and that such instruction be placed in the hands of experts. To which may be added, that the experts must really be experts, not merely assistant Poor-law medical officers told off for the business because their seniors cannot be bothered, and dignified with the name of experts for the purposes of departmental "eye-wash."

The next recommendation is much less judicious; in fact, it is downright stupid and unpractical. It is actually suggested that candidates for the C.M.B. certificates shall only be accepted on giving an undertaking to practice midwifery for not less than three years after qualification. This is illiberal, because its object is to diminish the number of candidates for C.M.B. certificates, and so to render available more clinical material for medical students. If the shortage of clinical material were absolute, there would be no need for this proviso, because there would be so few babies arriving that few women would want to take the C.M.B. certificate. But the shortage is not absolute; it is only relative and due to the fact that proper use is not made of the huge mass of clinical material which exists and is wasted. Besides being illiberal, the proposal is unpractical, because few nurses can bind themselves to follow one particular section of their art for three years ahead; even of those who are midwives alone, and not trained nurses, the pact is asking too much.

In conclusion, the Council draws the attention of recognised teaching institutions to the recommendations of the Council in 1906, in the hope that when the present difficulties in training are alleviated, they may be able to carry out these recommendations in their entirety, and that the present conditions of training, which in many cases cannot be approved, may be rendered such as the Council will be able to regard as satisfactory. This is plain speaking, but it will command the assent of most teachers and of most thoughtful members of the medical profession who look back on their days "on the district." There is no doubt that training in the lying-in ward, which only a small minority of the last generation of medical practitioners ever got, is a necessary part of proper instruction in this important branch of medical education; and it is to be hoped that the Council will eventually be successful in obtaining it and in being able to insist on it as part of the curriculum.

